# Roles of Epigenetics in Cardiac Fibroblast Activation and Fibrosis

**DOI:** 10.3390/cells11152347

**Published:** 2022-07-30

**Authors:** Jingrong Shao, Jiao Liu, Shengkai Zuo

**Affiliations:** 1 The Province and Ministry Co-Sponsored Collaborative Innovation Center for Medical Epigenetics, Tianjin Key Laboratory on Technologies Enabling Development of Clinical Therapeutics and Diagnostics, School of Pharmacy, Tianjin Medical University, Tianjin 300070, China; shaojingrong1997@tmu.edu.cn; 2 Tianjin Key Laboratory of Inflammatory Biology, Department of Pharmacology, School of Basic Medical Sciences, Tianjin Medical University, Tianjin 300070, China; drliujiao@tmu.edu.cn

**Keywords:** heart, fibrosis, epigenetics, fibroblasts

## Abstract

Cardiac fibrosis is a common pathophysiologic process associated with numerous cardiovascular diseases, resulting in cardiac dysfunction. Cardiac fibroblasts (CFs) play an important role in the production of the extracellular matrix and are the essential cell type in a quiescent state in a healthy heart. In response to diverse pathologic stress and environmental stress, resident CFs convert to activated fibroblasts, referred to as myofibroblasts, which produce more extracellular matrix, contributing to cardiac fibrosis. Although multiple molecular mechanisms are implicated in CFs activation and cardiac fibrosis, there is increasing evidence that epigenetic regulation plays a key role in this process. Epigenetics is a rapidly growing field in biology, and provides a modulated link between pathological stimuli and gene expression profiles, ultimately leading to corresponding pathological changes. Epigenetic modifications are mainly composed of three main categories: DNA methylation, histone modifications, and non-coding RNAs. This review focuses on recent advances regarding epigenetic regulation in cardiac fibrosis and highlights the effects of epigenetic modifications on CFs activation. Finally, we provide some perspectives and prospects for the study of epigenetic modifications and cardiac fibrosis.

## 1. Introduction

**Figure 1:**
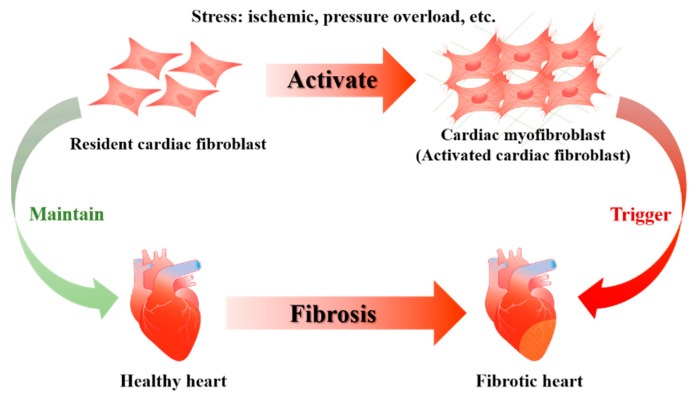
Cardiac fibroblasts activation and cardiac fibrosis. Activation of CFs into myofibroblasts is the cellular biological basis of cardiac fibrosis. Resident fibroblasts contribute to the maintenance of cardiac structure and homeostasis in a healthy heart. Under diverse pathologic stress and environmental stress, resident CFs in the injured heart are activated as myofibroblasts, infiltrate and proliferate in the injured area, synthesize and deposit excessive ECM, and eventually lead to the occurrence of cardiac fibrosis.

Cardiovascular disease continues to be the leading cause of morbidity and mortality worldwide [1]. Fibrosis, characterized by excessive deposition of extracellular matrix (ECM) proteins, occurs in many organ injuries and is related to many cardiovascular diseases [2]. Cardiac fibrosis, a common pathophysiological change in cardiac remodeling-related diseases, is a common pathophysiologic process in many cardiovascular diseases [3,4]. The main mechanisms of cardiac fibrosis are the excessive activation and proliferation of cardiac fibroblasts (CFs), which is a process that accompanies the progressive deposition of ECM [5,6]. The activated CFs, also known as myofibroblasts, are the major contributors to ECM production and major effector cells for fibrosis in the heart. Aberrant activation and proliferation of CFs are the cell biological basis of cardiac fibrosis [7]. Under physiological conditions, resident CFs are relatively quiescent and primarily responsible for maintaining extracellular matrix homeostasis, cardiac structure, and mediating electrophysiological conduction [8]. However, in the event of cardiac injury, such as ischemic injury and myocardial infarction (MI), myofibroblasts secrete more ECM and cause cardiac fibrosis [9,10] (Figure 1). Although there have been many studies on CFs activation, the molecular mechanism of fibrosis is still not well-understood.

Cardiac fibrosis is a very complex process regulated by multiple factors. A variety of studies show that epigenetic modifications have well-documented effects on the activation of CFs and the expression of fibrosis-associated genes [11,12,13]. Epigenetics differs from the realm of classical genetics by referring mainly to changes in DNA modifications (e.g., DNA methylation), protein modifications (e.g., post-translational histone modification), and RNA (e.g., non-coding RNAs and RNA methylation), without involving changes in genomic DNA base sequence [14,15,16]. Meanwhile, epigenetics also bridges genetics and environmental factors to explain genetic phenomena that genetics cannot. Actually, epigenetic modifications controlled by a series of writers (storing them), readers (interpreting them), and erasers (deleting them) are reversible [15,17]. The reversibility of epigenetics makes it easier for drugs to correct aberrant gene expression by modulating such regulators than by genetic alteration [11]. Meanwhile, diverse stimuli and stresses lead to changes in the epigenetic characteristics of CFs, which mediates the expression of pro-fibrotic and anti-fibrotic genes. Therefore, exploring the etiology of cardiac fibrosis from an epigenetic perspective will provide novel insight into fibrosis-targeting therapies. In this review, we summarized the function and underlying mechanisms of epigenetic modifications in CFs activation and cardiac fibrosis and highlighted epigenetic modifications as potential therapeutic targets for cardiac fibrosis-related diseases.

## 2. DNA Methylation in CFs Activation and Cardiac Fibrosis

### 2.1. DNA Methylation

One of the first and most extensively studied epigenetic modifications is DNA methylation, which is extensively involved in many biological regulatory processes such as gene expression regulations and chromatin structural changes [18]. In mammals, DNA methyltransferases (DNMTs) and demethylases cooperate to control DNA methylation levels; DNMTs transfer the methyl provided by S-adenosyl-L-methionine (SAM) to the fifth carbon atom of cytosine (C) to form 5-methylcytosine(5Mc) [18,19]. In general, DNA methylation is mostly modified on cytosine–phosphate–guanine (CPG) dinucleotides, which are mainly located in the promoter and exon regions of human genes [20]. DNA methylation is often associated with gene silencing, as it inhibits the interaction of chromatin to DNA-binding proteins or transcription factors (TFs) required for gene expression [21,22]. Growing evidence suggests that DNA methylation is involved in the cardiac fibrosis process through multiple pathways [11,23] (Table 1).

### 2.2. DNMTs in CFs Activation and Cardiac Fibrosis

DNA methylation modification consists of three phases: establishment, maintenance, and demethylation [18]. In mammals, DNMTs are responsible for catalyzing DNA methylation as writers. Although five DNMT proteins are found, only DNMT1, DNMT3a, and DNMT3b have catalytic methyltransferase activity. DNMT3a and DNMT3b are responsible for the de novo synthesis of methylation, and DNMT1 is responsible for the maintenance of the methylation state [37,38].

A recent study shows that DNMT3a inhibition prevents hypoxia-induced CFs activation and cardiac fibrosis [26]. In isoprenaline (ISO)-caused rat cardiac fi brosis model, DNMT3a promotes CFs activation and fibrosis through the ERK1/2 pathway [27]. Hedgehog (Shh) signaling is critical for the proliferation of CFs, and Patched1 is a negative regulator of the Hedgehog signaling pathway [39]. Interestingly, as the target gene of miR-369-5p, DNMT3a suppresses the Patched1 pathway by hypermethylation, promoting CFs proliferation in a cardiac fibrosis model of abdominal aortic stenosis [29]. Meanwhile, the pro-fibrotic effect of DNMT3a is also found to be associated with CFs activation and proliferation in the ISO-induced fibrosis model [28]. In addition, DNMT3a is also discovered to mediate fibroblast autophagy by regulation of miR-200b, which provides a new therapeutic strategy for cardiac fibrosis [30,40].

Like DNMT3a, DNMT3b is also thought to inhibit the hypoxia-induced CFs activation and fibrosis-related protein synthesis [31]. Methyl-seq analysis reveals aberrant methylation of the Ras protein activator like-1 (Rasal1) and Ras-association domain family 1 (Rassf1) promoters may be involved in the development of transverse aortic constriction (TAC)-induced cardiac fibrosis. *Salvia miltiorrhiza Burge* (Danshen) and *Carthamus tinctorius* are the medicinal herbs used for the treatment of cardiovascular diseases in Asian countries [41,42]. That Danhong injection (DHI), a Chinese herbal medicine composed of *Salvia miltiorrhiza* and *Carthamus tinctorius*, is found to prevent the hypermethylation of Rasal1 and Rassf1 through the downregulation of DNMT3b in CFs, which could reverse cardiac fibrosis and improve cardiac function [32,36].

Accumulating evidence has demonstrated that DNMT1 is involved in fibrotic disease [43,44]. In diabetic cardiac fibrosis, DNMT1 expression is increased in CFs. DNMT1-mediated SOCS3 promoter hypermethylation leading to SOCS3 axis silencing is a driver of CFs activation [24]. Furthermore, in ISO-induced cardiac fibrosis, DNMT1 methylation promotes the activation and proliferation of CFs by inhibiting the microRNA-152-3p via the Wnt1/β-catenin pathway [25]. Actually, methylation of DNA is not an isolated event but instead forges crosstalk. During myocardial hypoxia, HIF-1α-mediated ROS upregulates DNMT1 and DNMT3b expression and decreases Rassf1A expression through activation of Snail, which in turn increases the synthesis of related fibrosis markers [33].

**Figure 2:**
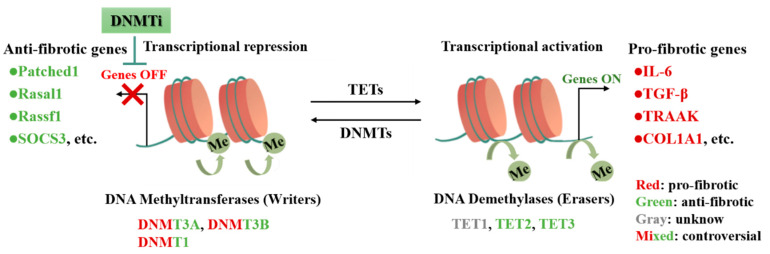
DNA methylation modifications regulate fibrosis-related genes involved in CFs activation and cardiac fibrosis. DNMTs and TETs cooperate to regulate DNA methylation levels, which determines the transcriptional activity of fibrotic genes as well as induces cardiac fibrosis. DNMTs as writers drive DNA methylation, repress transcription, and silence anti-fibrotic genes. Conversely, erasers’ TETs-mediated DNA demethylation activate transcription and involve in the activation of pro-fibrotic genes. As shown in the figure, different colors suggest that different genes and epigenetic-modifying enzymes play different roles in cardiac fibrosis. The application of DNMTi can target to alleviate DNA methylation-regulated cardiac fibrosis.

In summary, DNA methylation modifiers are implicated in the regulation of the critical processes of CFs activation and cardiac fibrosis [23,45]. Moreover, it is demonstrated that cardiac fibrosis is attenuated by modulating the readers or erasers influencing the DNA methylation process, such as DNMTs or the Ten-eleven translocation (TETs) [34,35,36], indicating that targeting such modulators may attenuate or reverse cardiac fibrosis. Nevertheless, as described above, different isoforms of DNMTs have different mechanisms in different models of cardiac fibrosis, making it impossible to precisely target therapy, and the regulatory process involves intricate signaling pathways and molecules with more side effects. Therefore, it is urgently needed to investigate new mechanisms of DNA methylation regulating cardiac fibrosis and to discover new regulatory pathways, such as mitochondrial metabolism [46], which may provide new strategies for the diagnosis and treatment of cardiac fibrosis (Figure 2).

## 3. Histone Modification in CFs Activation and Cardiac Fibrosis

### 3.1. Histone Modification

Histones are basic proteins with highly conserved sequences in the nucleus with functions in maintaining DNA structure, protecting genetic information, and regulating gene expression. Two copies each containing four core histones (H2A, H2B, H3, and H4) are organized as a histone octamer that wraps ~145 base pairs of DNA to form the basic unit of chromatin [47]. Histone modifications are an important component in epigenetic regulation as well as an important post-translational modification (PTM) pathway. Multiple histone modifications such as methylation, acetylation, phosphorylation, and monoubiquitination are performed at the NH2-terminal tails of different histones protruding from the nucleosome [11,48]. These modifications of histone amino acid residues not only affect the interaction of histones and other proteins, but also influence chromatin structure and gene expression [48,49]. Histone acetylation and histone methylation are the most deeply studied so far. Here, we will focus our review on the roles of these two epigenetic regulatory mechanisms in CFs activation and cardiac fibrosis.

### 3.2. Histone Acetylation in CFs Activation and Cardiac Fibrosis

Histone acetylation modifications mainly occur at the amino-terminal site of lysine, and the addition or removal of histone tail acetylation modification is a dynamic and reversible process, which is mediated by a family of histone acetylation “writers” and “erasers” including histone acetyltransferases (HATs) and histone deacetylases (HDACs) [50,51,52]. Histone acetylation “writers” and “erasers” modulate the sparseness of chromatin structure through site-specific acetylation modifications, which can precisely regulate gene expression. Specifically, HATs-mediated acetylation causes structural loosening of chromatin nucleosomes, promotes binding of TFs to genes, and enhances gene transcriptional activity; while HDACs-mediated deacetylation causes tight binding of histones to genes, inhibits transcription factors binding to promoters, and silences or suppresses transcription and expression of related genes [53,54]. HATs and HDACs work in concert to maintain the relative balance of histone acetylation modifications. HATs and HDACs are the nexus of multiple pro-fibrotic signaling networks that mediate the cardiac fibrosis process in multiple ways [55] (Table 2).

Epigenetic alterations caused by histone acetylation are closely associated with CFs activation, proliferation, excessive ECM deposition and cardiac pathological remodeling [72]. Acetyltransferase p300, a primary epigenetic writer of histone acetylation, is activated and promotes cardiac fibrosis via Smad2 in a high glucose-induced cardiac fibrosis model [56]. Type I collagen is epigenetically regulated by the acetyltransferase activity of p300 and its subsequent interaction with Smad3, and profibrotic cytokine TGF-β is unable to induce collagen synthesis in fibroblasts in absence of p300 [73,74,75]. Importantly, p300 inhibitors L002 and C646 could both reverse hypertension-induced cardiac hypertrophy and fibrosis in vivo [57,73]. In addition, curcumin, a natural product of p300-histone acetyltransferase inhibitor, suppresses the development of heart failure in vivo [76]. Moreover, curcumin analogues GO-Y030, a specific inhibitor of p300, is also reported to attenuate cardiac fibrosis in a mouse model of pressure overload [58]. Consequently, p300 holds promise as a specific target for anti-fibrosis. In addition, p300/CBP-associated factor (PCAF), a histone acetyltransferase, is also elucidated to be required for the activation of CFs in response to TGF-β1 [59]. Intriguingly, miR-134-5p knockdown protected myocardial remodeling and cardiac fibrosis in the rat model of MI by upregulating lysine acetyltransferase 7 expression and histone H3K14 acetylation [77].

Histone deacetylases HDACs are enzymes that catalyze the deacetylation of histone or non-histone lysine residues, known as “erasers”, which are necessary for the removal of specific PTMs from histones. HDACs are classified into four classes (I, II, III, and IV), of which classes I, II, and IV HDACs are deacetylated by a zinc-dependent mechanism, while the deacetylation of class III HDACs is mainly dependent on nicotinamide adenine dinucleotide (NAD^+)^ [78]. Class I HDACs include HDAC1, HDAC2, HDAC3, and HDAC8. Evidences from in vitro and in vivo studies indicate that class I HDACs are important regulators of cardiac fibrosis by CFs activation [79,80]. In-depth studies reveal that TGF-β may recruit HDAC1 to the Chloride channel accessory 2 (Clca2) promoter via Twist1, which inhibits Clca2 transcription by erasing histone H3/H4 acetylation. Subsequently, it is found that silencing of Clca2 promotes the activation of CFs into myofibroblasts and induces cardiac fibrosis, and Clca2 over-expression reverses this process and alleviates TGF-β-induced cardiac fibrosis [60]. Besides activating CFs, HDAC1 also functions to promote the proliferation and migration of CFs. A gene expression profiling study reveals peptidase inhibitor 16 (PI16) attenuates Ang-II-induced cardiac fibrosis by inhibiting HDAC1 expression [81]. Mechanically, PI16 attenuates Ang-II-induced elevated HDAC1 levels through the HDAC1/p53 signaling pathway, increasing H3K18 and H3K27 acetylation, thus inhibiting CFs proliferation and fibrosis-related protein expression, as well as significantly attenuating Ang-II-induced cardiac fibrosis [61]. Furthermore, inhibition of HDAC1 attenuates Ang-II-induced fibrosis by reducing mitochondrial overactivity and calcium overload [62].

In addition, HDAC2, HDAC3, and HDAC8 are also associated with cardiac fibrosis. HDAC2 may play a driving role in the development of cardiac fibrosis. HDAC2 activity is correlated with the phosphorylation status of HDAC2 S394 [82] and PPP2CA, the catalytic subunit of PP2A, which can bind to HDAC2 and inhibit HDAC2 function by preventing HDAC2 phosphorylation. Isoproterenol and pressure overload can induce cardiac fibrosis together with the process of PPP2CA and HDAC2 dissociation. Interestingly, ISO-induced cardiac hypertrophy and fibrosis are attenuated in PPP2CA transgenic mice with HDAC2 function inhibited [63]. It is noted that the depletion and inactivation of HDAC2 reduces Col1A1 and α-SMA expression in CFs [64]. Plantamajoside, an active component extracted from traditional Chinese herbal medicine *Plantaginis*, protects the myocardium from ISO-induced injury by inhibiting HDAC2 and AKT/GSK-3β signaling pathways [65]. Selective HDAC3 inhibitor RGFP966 significantly improves diabetes-induced cardiac dysfunction and fibrosis [66]. Furthermore, ISO treatment induces cardiac fibrosis by increasing the expression of HDAC8, p38 MAPK, and fibrosis-related genes. Using both the selective HDAC8 inhibitor PCI34051 [83] and the HDAC8 knockdown alleviate ISO-induced cardiac fibrosis by inhibiting the HDAC8-regulated p38 MAPK pathway [67].

Rhein, a Class I/II HDAC inhibitor, regulates fibrosis-related pathways and the cell cycle of CFs by inhibiting HDAC-dependent p53 protein stability under normoxic conditions [84]. The class II HDAC shuttles between the cytoplasm and the nucleus and is further divided into two subgroups: class IIa (HDAC 4, 5, 7, and 9) and class IIb (HDAC 6, 10). In myocyte-specific activated HDAC4 transgenic mice, activated HDAC4 promotes cardiac hypertrophy and fibrosis and exacerbates cardiac dysfunction in infarcted myocardium [68]. Moreover, sodium butyrate (NaBu) significantly attenuates Ang-II-induced cardiac hypertrophy and fibrosis, and inflammation by inhibiting the activation of the COX2/PGE_2_ pathway in HDAC5/HDAC6-dependent manner [69]. The other members of Class II HDACs have been sparsely studied in cardiac fibrosis, but have been discovered to be associated with other organ fibrosis models [85,86,87,88].

HDAC inhibitors (HDACIs) have shown strong promising potential for the treatment of cardiac fibrosis by inhibiting HDAC function [89]. The first-generation HDACIs (TSA, SAHA) have been shown to have anti-fibrosis properties; however, their molecular mechanisms remain to be elucidated. Currently, there are four HDACIs approved by the United States Food and Drug Administration (US FDA), all of which are pan-HDACIs, but none is applied to the treatment of fibrotic disease. Therefore, a comprehensive analysis suggests that there are still more barriers to the application of HDACIs in the clinical treatment of fibrotic diseases [90]. Firstly, during the development of fibrosis, there may be two or more HDACs exerting opposite effects simultaneously, pan-HDAC inhibitors may not achieve the expected effect or even have stronger side effects, and subtype-specific inhibitors should be developed to reduce adverse drug reactions. However, class I, II, and IV HDACs share a conserved catalytic domain and have very high homology among the same type of HDACs, making it difficult to develop subtype-specific inhibitors [91]. Secondly, both histones and non-histones are targets of HDACs, and many of them are key regulators of important pathways. Therefore, the regulation of fibrosis-related histone acetylation modifications may also cause strong toxicity [92]. Finally, the role of HDACs may differ at different stages of fibrosis development [93]. Consequently, a deeper understanding of the etiology of fibrosis is needed for more precise drug delivery to improve drug efficacy while reducing unwanted side effects.

**Figure 3:**
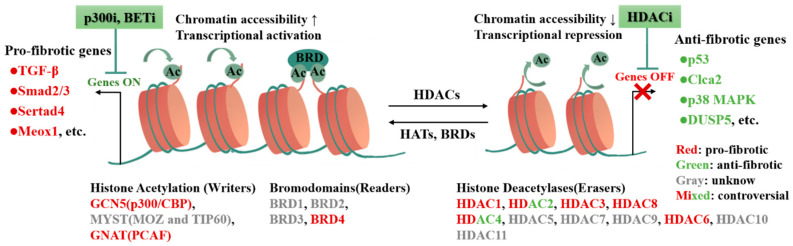
Histone acetylation modifications regulate fibrosis-related genes involved in CFs activation and cardiac fibrosis. The processes of histone acetylation and deacetylation are dynamically reversible, and histone acetylation modification states determine the chromatin accessibility as well as further transcriptional activity of fibrotic genes. HATs as writers and BRD4 as readers enhance chromatin accessibility and activate transcription, promoting pro-fibrotic gene expression. Conversely, HDACs mediate deacetylation as erasers, which reduce chromatin accessibility, repress transcription and participate in the epigenetic silencing of anti-fibrotic genes. As shown in the figure, different colors suggest different genes, and epigenetic-modifying enzymes play different roles in cardiac fibrosis. The application of p300i, BETi, and HDACi can target to alleviate histone acetylation-regulated cardiac fibrosis.

As highly conserved proteins recognizing and reading histone acetylation lysine modifications, the bromodomain-containing proteins (BRDs) play important roles in regulating gene expression [94]. Based on the structural or sequence similarity, BRDs are divided into eight families, among which the bromodomain and extra terminal domain (BET) protein families are most intensively and extensively studied [95]. BET inhibitor JQ1 reduces cardiac hypertrophy and fibrosis induced by phenylephrine or thoracic aortic fasciculation and heart failure after MI [96,97,98]. Moreover, BET inhibitor JQ1 also inhibits the expression of pro-inflammatory genes in CFs and suppresses CFs activation and proliferation in the DCM model. Inhibition of BET proteins abrogates adverse cardiac remodeling, reduces cardiac fibrosis, and prolongs survival. Mechanistically, BRD4, a member of the BET family, is a central regulator of the pro-fibrotic CFs phenotype through p38-dependent signaling. Inhibition of BRD4 suppresses the activation of CFs and alleviates cardiac fibrosis by silencing Sertad4 (SERTA domain-containing protein 4) and periostin (myofibroblast maker gene) [70]. In addition, the promising molecule C-34 exhibits effective BRD4 inhibitory activity. The novel BRD4 inhibitor C-34 silences the downstream target c-MYC and further inhibits the TGF-β1/Smad2/3 signaling pathway, alleviating CFs activation in vitro and cardiac fibrosis in vivo [71]. The anti-fibrotic effects of BET inhibitors are promising for alleviating cardiac dysfunction and fibrosis [99,100]. However, BET proteins also play an important role in maintaining myocardial contractile function and cellular mitochondrial homeostasis [101], and pan-BET inhibitors are likely to disrupt many other critical cellular functions. Therefore, a more in-depth dissecting of the precise molecular mechanisms of BET inhibitor regulation will facilitate precise targeting and reduce drug side effects. It is found that modulation of the transcriptional switch regulates CFs activation. In fibroblasts, the TF Mesenchyme homebox 1 (MEOX1) is a central regulator of stress-induced CFs activation and cardiac fibrosis. Blocking MEOX1 turn-on inhibits fibroblast activation, which plays similar roles with BET inhibitors. Mechanistically, BET inhibitors act as a reversible switch for fibroblast activation to precisely regulate MEOX1 turn-on in disease states, while selective CRISPR inhibition of the single most dynamic cis-element of the enhancer blocks TGF-β-induced MEOX1 activation and ameliorates cardiac fibrosis [9]. This study provides new perspectives on the plasticity and specificity of BET-dependent regulation in tissue fibroblasts and also provides new trans- and cis-targets for the treatment of fibrotic diseases [100]. Consequently, the development of drugs targeting BET will hopefully bring new light to the treatment of cardiac fibrosis [71] (Figure 3).

### 3.3. Histone Methylation in CFs Activation and Cardiac Fibrosis

Epigenetic regulation, especially histone methylation modification, is reported to be related to cardiac fibrosis [23]. Histone methylation modification is an important intracellular epigenetic modality that can regulate the transcriptional expression of specific genes by influencing chromosome conformation and the specific recruitment of downstream effector molecules. Histone methylation usually occurs at lysine (K) or arginine (R) residues in the tail of histone H3 and H4, and the modification site and number of methyl group all determine the transcriptional activity of the relevant genes [102,103]. Histone methylation can alter chromatin condensation to an open state that promotes chromatin transcription or to a closed state associated with decreased chromatin transcription. Histone methylation is catalyzed by histone methyltransferases (HMTs), which can be divided into protein lysine methyltransferases (KMTs) and protein arginine methyltransferases (PRMTs); and removed by histone lysine demethylase (KDMs), which are broadly divided into two families, Lysine-specific demethylase (LSD) and JMJC domain-containing family (JMJD). Methylation of histone lysine can exist in three states: monomethylated (me1), dimethylated (me2), and trimethylated (me3), which serve as markers of active or repressed gene expression [102]. Generally, H3K4me1/2/3 (short for mono/di/tri-methylation of lysine 4 of histone H3, similar below), H3K9me1, H3K27me1, H3K36me1/2/3, and H3k79me1/2/3 are considered to be active markers occupying actively transcribed gene regions in chromatin, while H3K9me3, H3K27me3, and H4K20me2/3 are known repressive markers that are usually associated with gene silencing and chromatin condensation [103,104]. KMTs and KDMs played important roles in cardiac fibrosis-related regulation (Table 3).

Based on the sequence of catalytic domains, KMTs can be classified into SET-containing domains and non-SET domains [116]. The SET domain is common to most transferases and is mainly responsible for the enzymatic activity of methyltransferases, including SUV39, EHMT1/2, EZH1/2, and RIZ (PRDM, SMYD, SUV420), and other families. Among them, enhancer of zeste homologue 2 (EZH2), a member of the EZH family, is most widely studied. EZH2 acts as the main catalytic subunit of the Polycomb repressive complex 2 (PRC2), which specifically catalyzes the methylation of histone H3 at lysine-27 (H3K27me1/me2/me3) through the SET structural domain [117]. EZH2 acts as an important epigenetic regulator during cardiac development and disease progression [117,118,119]. Interestingly, a high-fat diet during gestation or lactation in the mother induces cardiac reprogramming with reduced expression of EZH2 and DNMT3B and downregulates level of H3K27me2/3, which promotes the expression of pro-fibrotic and pro-hypertrophic genes [105]. In addition, EZH2 increases susceptibility to fibrosis in pathological processes [118], and that inhibition of EZH2 has the potential to reverse the fibrotic phenotype in cardiac disease. Both EZH2 inhibitor GSK126 and molecular silencing of EZH2 can block Ang-II-induced atrial fibroblast activation, migration, and ECM production in mice, attenuate Ang-II-induced atrial enlargement and fibrosis, and reduce atrial fibrillation vulnerability [106]. MiR-101a-3p might also prevent the development of atrial fibrillation in rats by targeting EZH2 to inhibit collagen synthesis and atrial fibrosis [120]. Similarly, miR-214-3p binds to EZH1/2 and represses EZH1/2 expression at the transcriptional level, which increases the peroxisome proliferator-activated receptor-γ (PPAR-γ) expression and inhibits Col1a1 and Col3a1 expression in myofibroblasts [109]. lncRNA-ANRIL alters the expression of fibronectin (FN), type IV collagen (Col1α4), and VEGF by interacting with multiple epigenetic regulators, including EZH2, p300, and regulates cardiac fibrosis in diabetic mice [107]. Furthermore, LncRNA NEAT1 inhibits Smad7 expression by recruiting EZH2 to the Smad7 promoter region, which ultimately aggravates the progression of cardiac fibrosis, while silencing NEAT1 significantly ameliorates TAC surgery-induced cardiac fibrosis and dysfunction in mice [108]. Moreover, in spontaneously hypertensive rats (SHRs), SUV39H1 is recruited by lncRNA MALAT1, resulting in H3K9me3 of MyoD-binding loci to mediate cardiac fibrosis [110]. Similarly, disruptor of telomeric silencing 1-like (DOT1L), the only non-SET domain KMT, promotes spleen tyrosine kinase (SYK) expression via increasing H3K79me2 modification of the SYK promoter, and upregulation of SYK activates TGF-β1/Smad3 signaling to mediate CFs proliferation and cardiac fibrosis. Knockdown of DOT1L significantly ameliorated the cardiac injury and fibrosis caused by MI [111].

**Figure 4:**
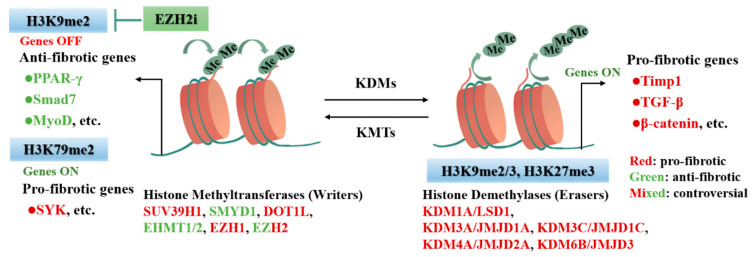
Histone methylation modifications regulate fibrosis-related genes involved in CFs activation and cardiac fibrosis. Histone methylation and demethylation are dynamic and reversible, and histone methylation modification sites and status determine chromosome accessibility and further transcriptional activity of fibrotic genes. Not only do writers KMTs drive H3K9me2, reducing chromatin accessibility and repressing transcription to silence anti-fibrotic genes, but they also drive H3K79me2, increasing chromatin accessibility, activating transcription, and promoting pro-fibrotic gene expression. Conversely, KDMs remove methyl from H3K9me2/3, which increases chromatin accessibility, promotes transcription, and is involved in the activation of pro-fibrotic genes. As shown in the figure, different colors suggest different genes, and epigenetic-modifying enzymes play different roles in cardiac fibrosis. The application of EZH2i can target the alleviation of histone methylation-regulated cardiac fibrosis.

Histone lysine demethylase (KDMs) is an emerging regulator of transcriptional reprogramming, though the potential mechanism in cardiac fibrosis is unclear. Currently, histone KDM is comprised of two subfamilies, Lysine-specific demethylase (LSD) and JMJC domain-containing family (JMJD) [121]. LSD family demethylases specifically remove mono- and di-methylated modifications of histone H3K4 and H3K9, while JMJD family demethylases catalyze the removal of three methylation modifications. LSD1/KDM1 has been recognized as an important target of cardiac fibrosis, whose direct substrates H3K4me1/2 and H3K9me1/2 play a critical role in cardiac remodeling [122,123]. Huo et al. reported that myofibroblast-specific LSD1 inducible knockout effectively alleviates cardiac hypertrophy, fibrosis, and pressure overload-induced heart failure by inhibiting the TGF-β signaling pathway [112]. KDM3A could remove H3K9me2/3 modifications, and the level of H3K9 methylation is downregulated in hypertrophic and failing hearts in mice and humans [124,125]. Tissue metalloproteinase inhibitor 1 (Timp1) is commonly used as a cardiac fibrosis biomarker, whose levels are associated with cardiac fibrosis in patients with heart disease [126]. In TAC-induced cardiac fibrosis, KDM3A binds the Timp1-promoter to remove methylation modifications and promotes TIMP1 transcriptional activation, which then activates CFs and enhances cardiac fibrosis [113]. Furthermore, TIMP1 is also identified as a potential target for KDM3C-mediated pro-fibrotic function. In the Ang-II-induced mouse model, KDM3C removes the methyl from the H3K9me2 on the Timp1 promoter, promotes Timp1 transcriptional activation, activates resident CFs, and induces cardiac fibrosis [114]. Furthermore, the H3K27me3 demethylase KDM6B removes the suppressive mark H3K27me3 from the β-catenin promoter in CFs, which activates CFs and significantly upregulates the expression of fibrogenic genes [115]. More studies are necessary to determine the effects of histone methylation regulators on the cardiac fibrosis process in pathophysiological situations [127]. Furtherly, we also need to understand the molecular mechanisms that include governing substrate specificity and the downstream effects of their catalytic activity (Figure 4).

## 4. RNA in CFs Activation and Cardiac Fibrosis

Epigenetic regulation occurs not only at the genomic level but also at the transcriptome level and plays an important role in post-transcriptional regulation [128]. RNA acts as the “bridge” between DNA and cellular protein production processes. Non-coding RNAs (ncRNAs) could regulate gene expression directly as epigenetic regulators or indirectly by modifying mRNAs and ncRNAs depending on regulators such as writers, erasers, and readers. Consequently, epigenetic modifications occurring at the transcriptomic level have critical regulatory roles in biological activities, and studies demonstrate that epigenetic modifications are closely associated with many diseases, including cardiovascular diseases (Table 4).

### 4.1. Non-Coding RNAs in CFs Activation and Cardiac Fibrosis

NcRNAs mainly include microRNAs (miRNAs), long non-coding RNAs (lncRNAs), and circular RNAs (circ RNAs), which are RNA transcripts with biological functions without protein-coding ability. Recently, there are growing evidences that ncRNAs can be involved in the development of cardiac fibrosis by influencing post-transcriptional levels of regulation of gene expression.

MiRNAs are short ncRNAs (<200 nucleotides) whose biological functions depend on complementary binding to the 3 ‘ or 5 ‘ -untranslated regions (UTRs) sequences of target mRNAs, inhibiting protein translation or targeting mRNA degradation to mediate post-transcriptional gene silencing. MicroRNA-369-5p (miR-369-5p) promotes fibroblast proliferation by inhibiting the Patched1 pathway through modulation of DNMT3A methylation modification [29]. Similarly, miR-489 also alleviates cardiac fibrosis by regulating HDAC2 to inhibit fibroblast activation and proliferation [64]. Interestingly, miRNAs not only act as upstream factors to regulate DNA methylation and histone modifications but also as downstream effectors to directly promote or inhibit fibrosis. It was found that miR-27b [129], miR-125b [130], miR-99b-3p [131], miR-143-3p [132], and other miRNAs regulate the activation, proliferation, and migration process of CFs by regulating the downstream fibrosis-related genes FBW7, Apelin, p53, GSK-3β, SPRY3, etc., facilitating the fibrosis development. The miR-21 in macrophages also regulates macrophage–fibroblast communication and promotes activation of quiescent fibroblasts into myofibroblasts, promoting cardiac fibrosis [133]. MiR-27b-3p suppresses mitochondrial oxidative phosphorylation (OXPHOS) through the target gene FGF1, and inhibition of FGF1 alleviates TAC or Ang-II-induced cardiac fibrosis by enhancing OXPHOS [134]. In addition, miRNAs such as miR-590-3p [135], miR-221/222 [136], and miR-1954 [137] alleviate cardiac fibrosis by regulating ZEB1, Smad2, and THBS1. SKI, a TGF-β negative regulator, promotes cardiac fibroblast deactivation and alleviates cardiac fibrosis [150]. It has been demonstrated that the epigenetic regulator miR-34a/miR-93 can affect both TGF-β and SKI and alleviate cardiac fibrosis. Mechanically, miR-34a/miR-93 affects TGF-β1-induced fibroblast proliferation and ECM deposition via SKI [151]. In addition, apigenin, a flavonoid isolated from *Apium graveolens*, shows promising protective effects against ISO-induced cardiac fibrosis. Apigenin alleviates cardiac fibrosis by upregulating miR-122-5p, c-Ski, and Smad7 and downregulating miR-155-5p, TGF-β1, hypoxia-inducible factor-1α (HIF-1α), Smad2/3, etc. [152].

LncRNAs as long noncoding RNAs (>200 nucleotides) are involved in the development of cardiac fibrosis mainly through directly binding to DNA, RNA, and proteins to regulate gene expression [128]. lncRNA AK081284 [138], lncRNA Meg3 [139], lncRNA Wisper [140], and lncRNA AK048087 [143] promote the activation and proliferation of CFs by regulating fibrosis-related genes such as IL-17, MMP-2, and COTL1. Meanwhile, lncRNA AK137033 regulates the stability of Sfrp2 mRNA [141], and lncRNA PCFL interacts with miR-378 [142], which together regulates cardiac fibrosis after MI. Furthermore, lncRNAs act as inhibitors to alleviate the development of cardiac fibrosis. LncRNA GAS5 overexpression can reduce TGF-β1-induced α-SMA and Col1A1 levels in CFs by inhibiting miR-21 expression to alleviate cardiac fibrosis [144]. LncRNA Crnde also suppresses CFs activation and alleviates DCM-associated cardiac fibrosis through negative feedback regulation of Smad3 transcriptional activation [145]. Dioscin, a glucoside saponin isolated from *Dioscorea nipponica Makino*, reduced MI-induced cardiac fibrosis by upregulating lncRNA MANTIS to promote the expression of angiogenesis-related genes such as SOX18, SMAD6, and COUP-TFII [146]. Meanwhile, histone demethylase JARID1B could also regulate lncRNA MANTIS and restrict the expression of angiogenesis-related genes such as SMAD6 [153]. However, whether JARID1B regulates lncRNA MANTIS through methylation modification and thus affects cardiac fibrosis remains to be further investigated.

### 4.2. RNA Modifications in CFs Activation and Cardiac Fibrosis

**Figure 5:**
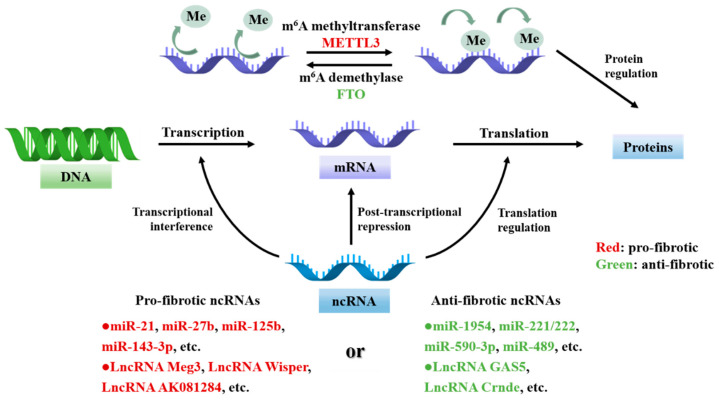
RNA regulates transcriptional and translational processes through multi-level ncRNA and RNA modifications involved in CFs activation and cardiac fibrosis. Multi-level of ncRNAs (e.g., miRNAs and lncRNAs) play cardiac pro-fibrotic and anti-fibrotic roles. In addition, m^6^A affects the expression of fiber-associated proteins by regulating the level of mRNA methylation. m^6^A modification process is dynamically reversible and co-regulated by METTL3 and FTO in CFs. As shown in the figure, different colors suggest that different ncRNA and epigenetic-modifying enzymes play different roles in cardiac fibrosis.

RNA modifications directly affect the biological functions of RNA. N6-methylated adenosine (m^6^A) is the most abundant epigenetic modification of eukaryotic mRNA, which is mainly involved in the pathophysiological process of cardiac fibrosis by regulating the transport, degradation, and translation of mRNA. m^6^A is regulated by three kinds of effector proteins, the m^6^A methyltransferase METTL3-METTL14 complex, m^6^A demethylases FTO and ALKBH5, and the m^6^A reader and the YTH structural domain proteins [154]. Overexpression of m6A methyltransferase METTL3 improves the level of m^6^A modification, increases collagen synthesis, and promotes cardiac fibrosis [147]. However, the expression level of m^6^A demethylase FTO is low in cardiac fibrosis induced by MI [155], hypoxia, and heart failure [149], and overexpression of FTO inhibits the activation, proliferation, and migration of cardiac fibroblasts and alleviates the development of cardiac fibrosis [148]. At present, the studies on m^6^A methylation in cardiac fibrosis are mainly focused on METTL3 and FTO. Future studies should investigate how other writers and erasers regulate the expression of downstream proteins, how m^6^A readers mediate the activity and mechanism of m^6^A methylation in CFs and further explore m^6^A methylation may provide new therapeutic strategies for cardiac fibrosis (Figure 5).

## 5. Conclusions and Future Prospects

In this review, we focus on the recent progress between epigenetic regulation and activation of CFs during cardiac fibrosis based on the main categories of epigenetic modifications. As key nodes, epigenetic-modifying enzymes can transmit upstream signaling through gene transcriptional profiling reprogramming to mediate cardiac fibrogenesis, providing a large number of potential drug targets for preclinical studies of cardiac fibrosis, and also providing a new perspective for the development of the clinical protocol in cardiac fibrosis [12,23]. It is important to elucidate the role of epigenetics in the mechanism of endogenous fibrosis, while it is equally necessary to focus on the studies of anti-fibrotic factors, especially those that involve the deactivation of cardiac myofibroblasts. Although there is currently rare research on the relationship between epigenetics and deactivation of cardiac myofibroblasts, future research in this area may yield some important breakthroughs due to the reversibility of epigenetic modifications. SKI, a negative regulator of the TGFβ1/Smad signaling, has a powerful ability to inhibit fibrosis and deactivate activated fibroblasts [150,156]. Studying the regulation mechanism of myofibroblasts deactivation, especially in epigenetic regulation, will also provide a new direction for the treatment of cardiac fibrosis. However, we must recognize that the regulatory processes of epigenetic modulators are extensive and complex. There is a crosstalk between each epigenetic modification, which can interact and influence each other. Meanwhile, complex feedback regulatory loops make it difficult to draw general conclusions about homogeneous epigenetic modifications, which increases the challenge of our epigenetics-based studies on the mechanisms of cardiac fibrosis.

Compared to genetic research, no genome-wide analysis of epigenetic modifications in CFs has been reported to provide a comprehensive and systematic understanding of the biological significance and catalytic activity regulation mechanisms of each epigenetic regulator. Nevertheless, with the development of gene sequencing technologies in recent years, the integration of single-cell RNA sequencing, single-cell epigenomics, high-resolution ChIP-seq, and HITS-CLIP (high-throughput sequencing with cross-linked immunoprecipitation) may provide a new approach to reveal the precise regulatory mechanisms, while the use of CRISPR-Cas9 gene-editing technology may also provide a more comprehensive resolution of epigenetic regulation. Meanwhile, several epigenetic modulator inhibitors, such as HDACIs, have been demonstrated to prevent and alleviate cardiac fibrosis. However, most inhibitors are not specific and may impair other key cellular functions during administration. Hence, more elaborate and comprehensive studies of epigenetic mechanisms are necessary to target more precise targets, thereby reducing the occurrence of adverse outcomes due to unnecessary inhibition or activation by drugs. In this process, it is also necessary to investigate whether and how epigenetic regulators exert cell-specific activities, which of course relies on further studies on the activation of CFs in cardiac fibrosis.

Growing evidences suggest that CFs activation is actually more complex than previously thought during cardiac fibrosis, and epigenetic modifications are very important for CFs activation and cardiac fibrosis. While many mechanisms need to be explored and conflicting evidence exists, future breakthroughs in the epigenetic field may provide avenues for the diagnosis and treatment of cardiac fibrosis. For example, chimeric antigen receptor T cell (CAR-T) therapy targeting FAP alleviates the degree of cardiac fibrosis and abnormal increased ventricular mass in model mice as well as improved cardiac systolic function, although the safety of this therapy needs to be further validated [157]. To better apply CAR-T to the treatment of cardiac fibrosis, researchers have also developed a way to generate transient CAR-T cells in vivo with mRNA molecules, which allows T cells to gain the ability to specifically target CFs for a short period to be fully effective and avoid affecting other cells [158]. Meanwhile, various means can reprogram CFs into cardiomyocytes, and epigenetics plays a key role in this process [159,160]; these advances will provide an alternative approach for clinical trials and treatments for patients with cardiac fibrosis in the future.

## Figures and Tables

**Table 1:** The roles of DNA methylation modifiers in CFs activation and cardiac fibrosis.

Subclass	Modifier	Fibrosis Model	Target	Output	Refs.
**DNA methylation**					
DNMTs	DNMT1	DCM	SOCS3	Pro-fibrotic, CFs activation	[24]
		ISO	microRNA- 152-3p	Pro-fibrotic, CFs activation and proliferation	[25]
	DNMT3a	TAC	TRAAK	Pro-fibrotic, CFs activation	[26]
		ISO	RASSF1A, ERK1/2	Pro-fibrotic, CFs activation	[27]
			Ras/ERK1/2	Pro-fibrotic, CFs activation and proliferation	[28]
		AAC	Patched1	Pro-fibrotic, CFs proliferation	[29]
			miR-200b	Pro-fibrotic, CFs autophagy	[30]
	DNMT3b	Hypoxia	HIF-1α	Pro-fibrotic, CFs activation	[31]
		TAC	Rasal1, Rassf1	Pro-fibrotic. CFs activation	[32]
	DNMT1, DNMT3b	Hypoxia	RASSF1A, ERK1/2	Pro-fibrotic, CFs proliferation	[33]
**DNA demethylation**					
TETs	TET2	Ang-II	IL-6	Anti-fibrotic, suppression of inflammatory response	[34]
		TET2 KO	Hspa1b	Anti-fibrotic, protection of cardiomyocytes	[35]
	TET3	TAC	Rasal1	Anti-fibrotic, EndMT	[36]

DCM, diabetic cardiomyopathy; ISO, isoproterenol; TAC, thoracic aortic constriction; AAC, abdominal aortic constriction; Ang-II, angiotensin II; CFs, cardiac fibroblasts.

**Table 2:** The roles of histone acetylation modifiers in CFs activation and cardiac fibrosis.

Subclass	Modifier	Fibrosis Model	Target	Output	Refs.
**Histone acetylation**					
HATs	p300	high glucose	Smad2	Pro-fibrotic, collagen production	[56]
		Ang-II	H3K9	Pro-fibrotic, CFs activation and type I collagen synthesis	[57]
		TAC	GATA4	Pro-fibrotic, collagen production	[58]
	PCAF	ISO	Smad2	Pro-fibrotic, CFs activation	[59]
**Histone deacetylation**					
HDACs Class I	HDAC1	LAD, TAC	Clca2	Pro-fibrotic, CFs activation	[60]
		Ang-II	p53	Pro-fibrotic, CFs activation and proliferation	[61]
			mitochondria	Pro-fibrotic, CFs migration	[62]
	HDAC2	ISO	PPP2CA	Pro-fibrotic, α-SMA synthesis	[63]
			α-SMA	Pro-fibrotic, CFs activation	[64]
			AKT/ GSK-3β	Pro-fibrotic, collagen production	[65]
	HDAC3	DCM	DUSP5	Pro-fibrotic, fibrosis markers and collagen accumulation	[66]
	HDAC8	ISO	p38 MAPK	Pro-fibrotic, markers of fibrosis	[67]
Class II	HDAC4	MI	N.A.	Pro-fibrotic, cardiokines reduction	[68]
	HDAC5/ HDAC6	Ang-II	COX2/ PGE_2_	Pro-fibrotic, cardiac hypertrophy	[69]
BRDs	BRD4	TAC	Sertad4	Pro-fibrotic, CFs activation and proliferation	[70]
		TAC	Meox1	Pro-fibrotic, CFs activation	[9]
			c-MYC	Pro-fibrotic, CFs activation	[71]

Ang-II, angiotensin II; ISO, isoproterenol; LAD, left-anterior descending; TAC, thoracic aortic constriction; DCM, diabetic cardiomyopathy; MI, myocardial infarction; N.A., not applicable. CFs, cardiac fibroblasts.

**Table 3:** The roles of histone methylation modifiers in CFs activation and cardiac fibrosis.

Subclass	Modifier	Fibrosis Model	Target	Output	Refs.
**Histone methylation**					
KMTs	EZH2	High-Fat	H3K27me2/3	Anti-fibrotic, suppression of pro-fibrotic genes	[105]
		Ang-II	ACTA2	Pro-fibrotic, CFs activation and migration	[106]
		DCM	lncRNA- ANRIL	Pro-fibrotic, increased expression of FN, Col1α4	[107]
		TAC	Smad7	Pro-fibrotic, CFs activation	[108]
	EZH1/2	Ang-II	PPAR-γ	Pro-fibrotic, Col1a1 and Col3a1 synthesis	[109]
	SUV39H1	SHRs	MyoD	Pro-fibrotic, CFs proliferation and collagen accumulation	[110]
	DOT1L	MI	SYK	Pro-fibrotic, CFs activation	[111]
**Histone demethylation**					
KDMs	LSD1	TAC	TGF-β	Pro-fibrotic, CFs activation and collagen secretion	[112]
	KDM3A		Timp1	Pro-fibrotic, CFs activation	[113]
	KDM3C	Ang-II	Timp1	Pro-fibrotic, CFs activation	[114]
	KDM6B		β-catenin	Pro-fibrotic, ECM deposition	[115]

Ang-II, angiotensin II; DCM, diabetic cardiomyopathy; TAC, thoracic aortic constriction; SHRs, spontaneously hypertensive rats; MI, myocardial infarction; CFs, cardiac fibroblasts.

**Table 4:** The roles of RNA in CFs activation and cardiac fibrosis.

Subclass	Modifier	Fibrosis Model	Target	Output	Refs.
**Non-coding RNAs**					
miRNAs	miR-27b	Ang-II	FBW7	pro-fibrotic, CFs proliferation and collagen production	[129]
	miR-125b		Apelin, p53	Pro-fibrotic, CFs proliferation	[130]
	miR-99b -3p		GSK-3β	Pro-fibrotic, CFs proliferation and migration	[131]
	miR-143 -3p	MI	SPRY3	Pro-fibrotic, CFs activation, proliferation, and migration	[132]
	miR-21	TAC	N.A.	Pro-fibrotic, CFs activation	[133]
	miR-27b -3p	TAC/ Ang-II	FGF1	Pro-fibrotic, mitochondrial oxidative phosphorylation	[134]
	miR-590 -3p	MI	ZEB1	Anti-fibrotic, CFs activation, proliferation, and migration	[135]
	miR-221/ 222	Ang-II	SMAD2	Anti-fibrotic, CFs activation, and proliferation	[136]
	miR-1954		THBS1	Anti-fibrotic, attenuation inflammation	[137]
lncRNAs	lncRNA AK081284	DCM	IL-17	Pro-fibrotic, CFs proliferation, and collagen production	[138]
	lncRNA Meg3	TAC	MMP-2	Pro-fibrotic, ECM deposition	[139]
	lncRNA Wisper	MI	TIA1-related protein	Pro-fibrotic, CFs proliferation	[140]
	lncRNA AK137033		Sfrp2	Pro-fibrotic, CFs activation, and proliferation	[141]
	lncRNA PCFL		miR-378	Pro-fibrotic, CFs proliferation, and collagen production	[142]
	lncRNA AK048087	MI/ Ang-II	COTL1	Pro-fibrotic, CFs activation, and proliferation	[143]
	lncRNA GAS5	ISO	miR-21	Anti-fibrotic, CFs proliferation	[144]
	lncRNA Crnde	DCM	Smad3	Anti-fibrotic, CFs activation	[145]
	lncRNA MANTIS	MI	Sox18, Smad6, etc.	Anti-fibrotic, vascular neogenesis	[146]
**RNA modifications**					
m^6^A	METTL3	MI	Fibrosis-related genes	Pro-fibrotic, CFs activation, and proliferation	[147]
	FTO	DCM	CD36, Slc5a33	Anti-fibrotic, collagen deposition suppression	[148]
		MI	Serca2a	Anti-fibrotic, CFs activation, proliferation, and migration	[149]

N.A., not applicable. DCM, diabetic cardiomyopathy; ISO, isoproterenol; TAC, thoracic aortic constriction; Ang-II, angiotensin II; MI, myocardial infarction; CFs, cardiac fibroblasts.

## Data Availability

Not applicable.
